# Comparative analysis of slenderization methods and their impact on enamel surfaces using SEM: An *in vitro* study

**DOI:** 10.6026/973206300213697

**Published:** 2025-10-31

**Authors:** Hrushikesh Aphale, Vinnayakk Kalaskar, Sunilkumar Nagmode, Vivek Shinde, Rahul Kolte, Vaishnavi Ingle

**Affiliations:** 1 Department of Orthodontics and Dentofacial Orthopedics, SMBT Dental college and Hospital, MUHS University, Sangamner -422608, Maharashtra, India

**Keywords:** IPR (Interproximal reduction), double-sided oscillating IPR strips, interproximal stripping burs, carborundum discs, scanning electron microscopy (SEM)

## Abstract

Interproximal reduction (IPR) is an orthodontic technique that removes 0.3-0.5 mm of enamel from interproximal surfaces to create space, typically for mild to moderate crowding. It helps reshape approximal contacts and address Bolton's discrepancy. Various methods, including air rotor stripping, diamond-coated disks and abrasive strips, are used for IPR. This study compares three IPR techniques Double-sided oscillating IPR strips, interproximal stripping burs and carborundum discs-by examining their effects on enamel surfaces using scanning electron microscopy (SEM).

## Background:

In several clinical situations, interproximal reduction, or IPR, is commonly used in orthodontic therapy [1]. Reduction of interdental gingival papilla retraction, mild to severe crowding, morphologic dental abnormalities, as well as correction of Bolton tooth size discrepancies are the primary clinical indications [1, 2, 3, 4-5]. It is commonly employed in combination with clear aligners as part of treatment [6]. Sheridan introduced the Air-rotor stripping (ARS) technique over 20 years ago as an alternative to extraction borderline situations. Over the years, several IPR systems have been created and gradually altered. Abrasive strips, diamond-coated segmented discs, as well as mechanical oscillating IPR devices have all become more popular recently [6, 7- 8]. As the use of IPR techniques in orthodontics has increased, numerous researches have examined the impact these procedures have on the surface of enamel [9, 10]. Qualitative SEM analyses revealed that IPR systems can alter the morphology of enamel, causing scratches and furrows [6, 9, 10-11]. In 1956, Hudson [17] developed employing medium as well as fine manual metallic strips, followed by polishing along with topical fluoride application to decrease enamel abnormalities [12, 13]. To improve enamel remineralization following IPR, Bonetti *et al.* recommended topical administrations of casein phosphopeptide amorphous calcium phosphate [14]. Only a small number of studies examined the effectiveness of the several IPR systems now in use [8, 15, 16-17]. Lapenaite *et al.* examined various IPR systems, emphasizing their indications, consequences along with contraindications in a recent literature study [7]. Although there are clear variations in efficiency, impacts on enamel surface roughness, as well as technical factors like application speed, abrasive grain size, as well as intensity of usage, instruments are successful in lowering interproximal enamel. Quantifying the amount of enamel that can be removed is also crucial to avoid inter-arch disparities, excessive space and persistently misaligned teeth. Achieving treatment goals requires high accuracy, particularly when using 3D digital treatment plans [18]. Mechanical oscillating abrasive strips have become the most popular IPR system due to their accuracy, effectiveness, reduced chairside time and minimal enamel damage. Studies comparing mechanical oscillating and manual diamond strips have shown the former to be more efficient in enamel reduction and surface uniformity. Additionally, the polishing phase after IPR enhances enamel smoothness [6, 7]. A standardized clinical sequence should be followed for IPR, including interproximal opening, enamel removal, checking enamel reduction and finishing/polishing. Various stripping methods aim to achieve smoother surfaces, with ongoing improvements to refine techniques. Understanding the abrasive lifespan of IPR systems is crucial for maintaining efficiency and ensuring optimal orthodontic outcomes [5, 6 and 7]. Therefore, it is of interest to assess the manual and mechanical techniques with claims of a smoother post-reduction enamel surface that is amenable to remineralization. This study compares three IPR techniques Double-sided oscillating IPR strips, interproximal stripping burs and carborundum discs-by examining their effects on enamel surfaces using scanning electron microscopy (SEM).

## Methodology:

This is a cross-sectional prospective *in vitro* study conducted in Department of Orthodontics and Dentofacial Orthopedics. This *in vitro* study used 15 sets of paired teeth for slenderization procedures, mounted on an acrylic model. Three techniques-IPR burs, carborundum discs and double-sided IPR abrasive strips-were tested comprising 3 groups. Each group consists of 5 samples. The sampling technique selected was Purposive sampling technique. A sample of anterior teeth and premolars was gathered to mount the teeth on acrylic mould. All 15 samples in total undergo 3 comparative IPR techniques including IPR burs (5), IPR double-sided strips (5), IPR carborundum discs (5). IPR Time for each technique was recorded with a standardized 20-second digital stopwatch. IPR burs and carborundum discs were operated with their respective handpieces and double-sided IPR strip was operated with IPR handle manually by the operator. Each method was tested for 8cycles (30s each) for the 3IPR procedures, to track the amount of enamel lost over the course of 8cycles. The efficiency of enamel reduction was first assessed by contrasting the amount of enamel reduction that 3 IPR systems could accomplish in a predetermined period. The experimental analysis was performed utilizing JEOL Model S7000 SEM and Insize C002 contact profilometer to analyse the objectives of the study and the loss in burr diamond crystals. A contra-angle was used in the experimental setup to achieve the reciprocating movement. Each strip, IPR bur and carborundum disc underwent 1 test consisting of eight cycles. The effects on strips', IPR bur and carborundum disc surface structure were analyzed employing SEM. Specifically, abrasive tracks were examined to assess the distribution of abrasive grains on metallic strip matrix as well as even existence of enamel debris both before as well as during use SEM evaluation. Examining the data of enamel reduction from 8cycle testing allowed for the investigation of abrasive property deterioration for both IPR systems. Briefly, the objectives of the study are mentioned below in Figure 1 (see PDF).

## Results:

The results from the above tables show significant statistical values. The following data was calculated through One-way Anova Test and pairwise comparison was done with Posthock Tuckey's test. From Table 1 (see PDF) it is appreciated that enamel loss was more with using carborundum disc followed by IPR bur. the IPR strip caused less enamel loss. Pairwise comparison showed a greater mean difference when the disc was compared with IPR disc and with IPR bur, which showed statistically significant data. Table 2 (see PDF) represented the abrasive decay of IPR strip to be less than compared to IPR disc and IPR bur. IPR strip was found to be more efficient for interproximal stripping procedure with better abrasive lifespan of IPR system ensuring optimal efficiency, allowing timely replacement for better orthodontic results. These results also showed statistically significant data outcomes for abrasive decay parameter. Table 3 (see PDF) depicts the surface roughness between the three comparing groups which showed IPR strip showed less surface roughness and IPR disc showed higher surface roughness between the three groups followed by IPR bur.

## Discussion:

To optimize occlusion, manage space and improve alignment, a common orthodontic method called interproximal reduction (IPR) aims to decrease the mesiodistal tooth width. Several IPR systems, such as rotating diamond burs, diamond-coated segmented disks, mechanical oscillating abrasive systems and manual abrasive strips, have been developed in response to the growing desire for alternatives to tooth extraction [1, 2, 3, 4, 5, 6, 7-8]. There are still worries about IPR's possible effects on enamel integrity, even though it has several clinical benefits. The impacts of three IPR techniques on enamel surfaces are assessed in this work using contact profilometry and scanning electron microscopy (SEM): mechanical reduction using diamond IPR burs, manual stripping using abrasive strips and rotary carborundum disc systems. All of the stripping techniques considerably roughened the enamel surfaces, according to Bonetti *et al.* and Arman *et al.* However, no studies in literature analyzed the effects of different IPR systems with their abrasive decay and loss of enamel volume on biological structures such as teeth [1, 6, 7- 8, 13]. Numerous researches has examined the impact of various IPR techniques on the enamel surface as they have become more common in orthodontic practice. Following both IPR methods, the enamel surface in this research looked rougher than the untreated control. In comparison to the double-sided manual IPR strip, mechanical IPR systems, IPR burs and carborundum discs produced more uniform enamel surfaces with effective tooth-cutting efficiency, but they also displayed higher levels of surface roughness. Qualitative SEM evaluations revealed that these discs can alter enamel morphology, leaving scratches and furrows that have the lowest scores for surface roughness and waviness measures. Findings show that, in comparison to manual abrasive strips, Livas *et al.* mechanical IPR systems, which include burs and carborundum discs, generate a more uniform surface with light parallel lines and tiny grooves [7]. However, compared to manual IPR double-sided strips, the current study revealed that IPR burs and carborundum discs had more roughened surfaces with irregular fragments, extended groves, as well as enamel ridges. This suggests that while the single use of metallic abrasive strips may be effective for enamel reduction, it isn't considered a biological structure. In line with the qualitative assessment, surface roughness values demonstrated a declining trend following the sequence and more uniform surfaces free of blemishes and imperfections. Because of the decreased precision and great variability of the abrasive grain size as well as distribution, enamel defects were most noticeable. The grain size of abrasive particles has a major impact on both the achievable enamel surface quality and the effectiveness of dental abrasives [4, 5]. The three IPR systems' abrasive particle grain sizes varied, according to the SEM analysis. In these investigations, the mean particle size of mechanical IPR systems was around 80 µm, while the grains on manual abrasive strips varied in size. With an accuracy of 0.1 mm-a prerequisite for 3D treatment plans like clear aligners-the mechanical IPR system (IPR burs and Carborundum IPR disc) showed superior enamel reduction efficiency. The manual IPR system removed an average of 0.09 mm throughout the eight test cycles, whereas the mechanical IPR system (IPR bur and carborundum disc) removed more enamel than the manual IPR system (IPR strip) [6, 8]. While a steady decline was seen for both systems, the percentage of enamel degradation during the eight cycles for mechanical IPR systems was noticeably higher due to abrasive property decay. Even though the cutting efficiency was lower than that of IPR bur and carborundum disc, manual double-sided IPR strips demonstrated reduced abrasive deterioration, making them more effective. Gazzani *et al.* claimed that enamel debris and abrasive grain separation were responsible for the gradual loss of abrasive qualities. A qualitative examination of abrasive surfaces following cycles of test revealed that these impacts were less noticeable on IPR burs and carborundum IPR discs [17].

## SEM analysis:

SEM analysis of tooth under carborundum disc showed grooving and chipping of tooth surfaces (Figure 2 - see PDF) with higher average value for waviness shown in Figure 2A (see PDF). Following this mechanical IPR process, the enamel had a more uneven surface with long ridges, groves and irregular pieces that suggested some inconsistencies in the hand abrasive track from Figure 2B (see PDF). Figure 3 (see PDF) Following SEM analysis for IPR bur used on the tooth surfaces showed grinding with mechanical oscillating systems (IPR bur) resulted in rougher enamel surfaces in comparison to untreated ones with mean average value for waviness lowest of all the three IPR techniques. Figure 4 (see PDF) This SEM analysis shows the impact of double-sided IPR strip on the tooth showing indents and grooving as observed for the carborundum disc. Chipping of tooth surface was also evident. The average waviness was seen to be increased but lower than carborundum disc. Distinct shapes as well as sizes of the incisions made on the tooth by three distinct IPR systems before and after IPR were revealed by the SEM analysis of the IPR bur (Figure 3A - see PDF and B - see PDF), carborundum disc (Figure 2A - see PDF and B - see PDF) and double-sided IPR strip (Figure 4A - see PDF and B - see PDF) following IPR. Manual and mechanical IPR systems presented abrasive grains of variable dimensions and grain shapes. The surface was continuous without holes before IPR. All three IPR systems show abrasive grains with irregular dimensions of abrasive grains with scratches and furrows. Regarding the abrasive property deterioration, while a consistent decline was noted for all three methods, percent of enamel reduction throughout the eight cycles was considerably lower for double-sided IPR strips than for IPR burs and carborundum discs. The enamel debris on the strip surface and the separation of some abrasive grains following slenderization are the causes of this steady loss of abrasive qualities. The presence of enamel debris was more seen in the IPR bur and carborundum disc when compared to manual IPR strip technique but showed greater enamel cutting efficiency when compared with IPR strips. After the test cycles, the qualitative evaluation of the abrasive surfaces showed that these two occurrences were less noticeable on IPR strips.

## Conclusion:

The present study demonstrates that double-sided strips yielded the smoothest enamel, followed by burs, with carborundum discs producing the greatest roughness. Mechanical methods created more uniform surfaces but caused greater enamel loss, while manual strips showed more irregular surfaces. Strips and burs maintained abrasive efficiency longer than discs, with diamond strips and rinsing improving polishing and clinical outcomes.
